# Domain randomization for neural network classification

**DOI:** 10.1186/s40537-021-00455-5

**Published:** 2021-07-02

**Authors:** Svetozar Zarko Valtchev, Jianhong Wu

**Affiliations:** Laboratory of Industrial and Applied Mathematics, York University, 4700 Keele St, M3J 1P3 Toronto, ON Canada

**Keywords:** Domain randomization, Synthetic image generation, Neural network classifiers

## Abstract

Large data requirements are often the main hurdle in training neural networks. Convolutional neural network (CNN) classifiers in particular require tens of thousands of pre-labeled images per category to approach human-level accuracy, while often failing to generalized to out-of-domain test sets. The acquisition and labelling of such datasets is often an expensive, time consuming and tedious task in practice. Synthetic data provides a cheap and efficient solution to assemble such large datasets. Using domain randomization (DR), we show that a sufficiently well generated synthetic image dataset can be used to train a neural network classifier that rivals state-of-the-art models trained on real datasets, achieving accuracy levels as high as 88% on a baseline cats vs dogs classification task. We show that the most important domain randomization parameter is a large variety of subjects, while secondary parameters such as lighting and textures are found to be less significant to the model accuracy. Our results also provide evidence to suggest that models trained on domain randomized images transfer to new domains better than those trained on real photos. Model performance appears to remain stable as the number of categories increases.

## Significance statement

We set out to see if strategic image synthesis could be used to create training data for an image classifier, and how well it would stack up against a classifier trained on real world images. Our results suggest that domain randomization can create datasets that produce competitively accurate classifiers, while also having the added benefit of transferring better to new unseen domains. These results are highly promising since generating synthetic data is inexpensive and rather quick, as oppose to creating datasets from scratch for every new class that doesn’t exist yet. This would substantially lower the barrier to entry into AI for people all around the world, providing unbiased and perfectly annotated data, while also leading to drastic speedups in computer vision research.

## Introduction

Recent advances in convolutional based neural networks have opened the door for automation in a wide variety of visual tasks, including classification, segmentation, object tracking, viewpoint estimation and view synthesis. Where once the challenge was to develop a strong model for each of these tasks, today the challenge has jumped to the acquisition of large and diverse datasets to train the models. Zero-shot and few-shot learning has been making progress in tackling the data size requirement, but solutions there are still very case dependant. They also still have problems with black swan events [1].

The cost of gathering a large amount of data can be very expensive, both in terms of money and time. Pixel level segmentation on an image, for example, can take hours to properly label on a complex enough scene. Instead, the task can be offloaded to a computer, not only in synthesizing the visual data, but also in annotating it [2–6]. In this fashion, large datasets can be produced relatively quick and cheap, and any labelling comes not only free, but at beyond human-level accuracy.

The use of synthetic data however introduces what is known as the reality gap [7], which as the name suggests, is the inability for it to fully generate to the real world data, for numerous reasons including textures, lighting and domain distributions. Achieving photorealism in the synthetic data, comes at the price of computational resources and render time [8]. Ray tracing engines can produce images indistinguishable to the untrained eye from a real photo, but may take dozens of hours to render a single image. Instead, realtime renderers like those used by popular game engines are usually used due to their ability to produce large datasets quickly.

In an attempt to narrow the reality gap, domain randomization is introduced to simulate a sufficiently large amount of variations such that real world data is viewed as simply another domain variation [7, 9]. This can include randomization of view angles, textures, shapes, shaders, camera effects, scaling and many other parameters.

Domain randomization has been successfully shown to aid in the training of networks for object detection, image segmentation, spatial positioning and depth estimation. For our purposes, we will aim to study it’s effectivity specifically to image classification. We further perform a collection of univariate tests in order to (1) examine which parameters are most significant to the process, and (2) gauge the resulting model’s ability to generalized to new domains.

## Related work

To address the reality gap, domain randomization techniques have been explored, including most notably the work of Tobin et al. [7], where they synthesized images of basic geometric objects on a table, in an attempt to estimate their spacial coordinates, such that a robotic arm could pick them up. Their accuracy varied depending on domain parameters, achieving errors as low as 1.5 cm on average in terms of object location, showing promise for synthetic data training. Notably, they found that the number of images and the number of unique textures used in the images were the most prominent parameters to model accuracy. Camera positioning and occlusion also had meaningful contributions, while the addition of random noise in images did not.

In another work, Tobin [9] discussed the use of the domain randomization, to the objects themselves, again in the aim of robotic grasping. This time, they procedurally generated millions of object meshes, leading to shapes not typically seen in the physical world, with the goal of the model generalizing specifically to the motion of the grasp. When bridged with real world objects, their results show the robot is able to grasp with an 80% accuracy. Again, the number of objects was a contributing factor to the overall accuracy.

Loquercio et al. [10] used domain randomization to bridge the gap between the artificial world and the real one, in the task of autonomous drone flight. In their work, they synthesized arbitrary race courses for the drone to learn to fly in, and then tested their controller in arbitrary track configurations in the real world. They achieved near perfect course completion scores for many variations including max speed constraints up to 10 m/s, and lap totals less than 3. Other results are also substantially higher than other baselines they compared to. Parameters tuned include scene textures, gate shapes, and lighting conditions, with all 3 providing improved results. Similarly, Shafaei [3], and Atapour-Abarghouei [4] showed that depth estimation in general can be well approximated using neural networks trained entirely on synthetic images. They do note however, that having pixel-perfect annotations lead to problems when generalized to a real world domain.

Tremblay et al. [2] outlined a variety of different parameters associated with the domain randomization process in the problem of object detection, with results indicating more parameters give rise to better accuracy, even if only marginally. Furthermore, they noted that freezing the weights of the feature extractor part of the network resulted in worse results, which contrast results obtained by Hinterstoisser [11]. Peng [12] also highlighted this notion, by lowering their learning rate, as to further allow the feature extractor itself to generalize to higher level features, which may only be present in the synthetic data. Their results indicated that viewpoint variation of the objects is far less important in terms of model accuracy than one might suspect, while the model is most sensitive to the amount of unique instances of objects per class.

Another common computer visual task is the problem of viewpoint estimation. Movshovitz-Attias [8] explored this using carefully constructed synthetic images, leading to results nearly as good as real images.

A natural place to utilize synthetic images is in the problem of human pose estimation. Rigging of joints is an expensive and cumbersome tasks, and generating large datasets for this are nearly impossible. Chen et al. [13] show that training with synthetic images for the task, outperformed training on real images.

Pepik [14] highlighted how convolutional neural network accuracy doesn’t necessarily transfer across different domains, providing evidence that the networks are not invariant to domain changes. They concluded that training them with mixed data spread across many domains increases generalized performance, similar to the findings of Peng et al. [15].

Improvements to regular domain randomization have been shown when the sampling methods and parameter distribution is carefully adjusted as show by Mozian et al. [16] and Mehta et al. [17]. Prakash et al. [18] were able to apply such methods successfully in the 2D bounding box problem. The team at OpenAI managed to train a robotic hand to solve a Rubik’s Cube by generating progressively more difficult environments in what they called automatic domain randomization, effectively updating their sampling during training [19].

The common findings between most previous research into synthetic data appears to be that it is best used as a supplementary part of the data gathering process, as oppose to entirely relying on it to train a model from scratch. Generally, a mixture of real world data, spread across a broad range of domains, in unison with computer generated imagery tends to produce the best results [20].

Literature in domain randomization for image classification is lacking. We aim to address this shortcomings, and apply similar analysis to it’s application in terms of accuracy to real world data, as well as it’s transferability to other domains. We also explore the effects of different domain randomization parameters in Sect. "Domain Randomization Parameters".

## Methods/experimental

To generate our images we make use of popular game engine Unity. We make this choice as we aim to produce a large amount of visual data at a reasonable tradeoff relative to the rendering time required. A game engine in particular is great versus photo-rendering software since it can produce many frames per second, at the cost of photorealism. Since one of the central inspirations to using synthetic data is the fact we can generate lots of data quickly, it does not make sense to spend hours rendering each image to cinematic quality, as this may be even more costly than gathering the real image data by hand.

Domain randomization aims to produce data samples from a large variation of the possible image space. The samples must not follow any real-world observed distribution, hence possibly producing just as many extreme real-world outliers. Consider, for example, how an autonomous car driving model will perform when it comes across a car accident on the road, having been trained only on data of clean law-abiding agents. For our classifier, we aim to train our model to detect precisely what features identify each class, regardless of how feasible each sample is.

For our analysis, we begin by importing 3D generated models of cats and dogs from the Unity Asset Store. We proceed to build a random scene with one of these models rendered each frame. First a plane is created, on top of which a model of one of our classes is placed. Our models come rigged with a variety of different animations, and so we randomly sample a frame from a random animation for each subject. This gives us a variety of different poses, sampling across what we can think of as a posture manifold across image space. Similarly, any other simulation parameter can be interpreted as distinct manifold, allowing for sampling across samples that should be invariant in terms of classification. Such sampling should encourage our network to generalize better to the specific features that define each class, as oppose to other latent features. Next we place our camera at a random position in the upper hemisphere of the plane, keeping a reasonable maximum distance to the subject. The camera is rotated towards the subject with slight perturbations to simulate random locations on the rendered image. Lighting of the scene is randomized in terms of intensity and direction, and a variety of skyboxes were used to generate a random sky. Random 3D volumes are spawned near the subject, at random scales, locations and orientations. We limit the amount of occlusion these volumes can have in frame as not to produce any samples where the entire subject is hidden. Lastly all objects in the rendered viewpoint are given a random texture. For our data generations, we used 74 different materials including rocks, woods, metals and even unrealistic textures such as sprinkles. We refer to all of these augmentations as the parameters of the synthesizer. Other augmentations such as brightness and saturation adjustments, noise addition and image deformation are excluded from the setup, as they are often done at train time anyway.

**Fig. 1:**
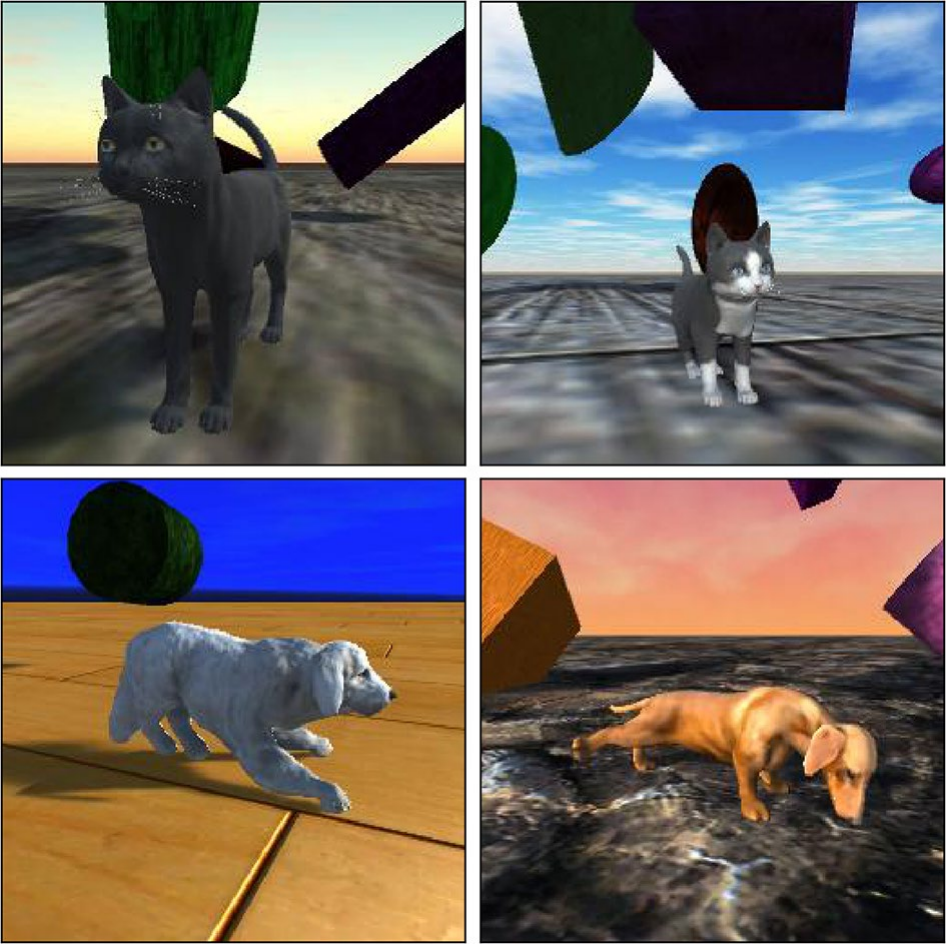
Examples of synthetic data generated using domain randomization of cats and dogs. Materials, breeds, skyboxes, camera, lighting, pose and obstructors are randomized across images

The entire scene described above is generated every frame and a $256 \times 256$ snapshot is save in JPEG format. Examples can be seen in Fig. 1. Our current setup runs around 20 frames per second, making the image synthesis very efficient, as it allows us to generate 1000 images in less than a minute.

When we refer to randomization in our simulation, we consider a uniform distribution for our subsampling. For example, any location of the camera is equally likely to be chosen, and so we inherently achieve a flat distribution which may not be present in a real world training set. Photographers tend to capture images in canonical views and so this may produce a very bias sample with many head-on and profile views, and not much in between. By using a uniform distribution, we encourage the network to learn the general structure of each class, as oppose to possibly overtraining it to a specific viewpoint, and failing to generalize in cases where a novel viewpoint is encountered. We apply this across all simulated parameters.

## Results

Under the conditions that synthetic data is cheaper and easier to obtain than authentic real world data, we are interested in the quality of such data. More precisely we want to study the effect of synthetic data on the overall accuracy of a convolutional classifier. In order to have a fair comparison, we will keep the number of training samples the same. We will also use the same real world test data for both.

**Fig. 2:**
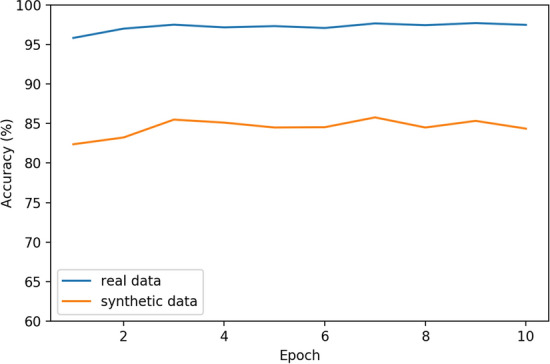
Test accuracy of the model using real training data vs synthetic training data over 10 epochs

We use the Kaggle Dogs and Cats Dataset [21] to gather 10,000 train images and 2500 test images for dogs and cats each, for a standard 80–20 split. We also proceed to generate 10,000 synthetic images of each category as well using the methods outlined in Sect. "Methods/experimental". In order to keep network architecture choices from effecting our results, we will proceed with a basic pre-trained VGG-16 model and stack one 128-neuron layer on the end of it before making a final classification. This way all feature extraction is done by a standard visual feature extractor and the test is not biased only to the task at hand. Standard data augmentation techniques are applied on images at train time, including random cropping, zooming, horizontal flipping, and slight rotations and brightness adjustments. Training the network on a single NVIDIA Tesla K80, with a learning rate of 0.001 over 10 epochs, produces the results in Fig. 2. Since we have a large set of training data, our model converges quickly. We can notice that the real model achieves a state-of-the-art accuracy of near 97.5% while the synthetically trained model underperforms at 86%.

### Network architecture

**Fig. 3:**
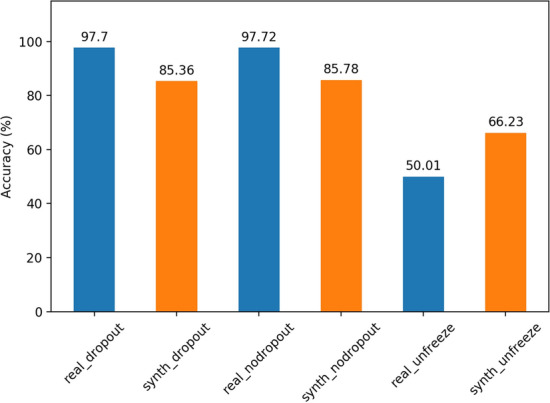
Accuracy of the real and synthetically trained models using dropout, no dropout, and allowing for the VGG weights to be trainable (unfreezing)

Since the inspiration behind domain randomization relies on the idea of model generalization across different domains, it may be of interest to see how the model accuracy will be effected by implementing dropout into the network (0.3 dropout rate), as well as unfreezing the weights of the VGG-16 part of the network as mentioned in [2] to allow for full model finetuning. Results of optimal accuracy through 10 epochs of training for each can be seen in Fig. 3. Dropout seems not to have any noticeable effects on the accuracy of the model, while unfreezing the VGG-16 weights seems to have reduced accuracy substantially, in line with results from [11] and [12] suggesting that only the final layer should be finetuned in practice. This could likely be explained by the fact that the VGG network is very large and has taken tons of computational power to train, whereas we only terminate training after 10 epochs. This can be explored further, but will likely require large amounts of resources.

### Domain randomization parameters

It would be valuable to know which parameters in our domain randomization are most important in the quality of the data, and to what degree. To study this, we train the same model above on a variety of different synthetic training data sets. Each set varies only in the inclusion/exclusion of a domain randomization parameter, while keeping all others present as the control case in Sect. "Network architecture". This way we can gauge the importance of each parameter in its overall quality contributions to the model accuracy.

**Fig. 4:**
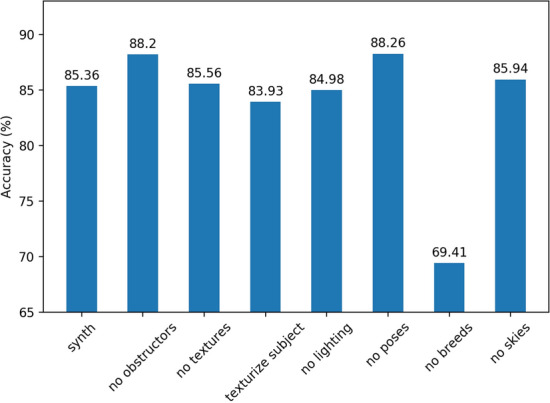
Optimal accuracy of models trained using different synthetic datasets. “Synth” represents the control model trained on domain randomization techniques outlined in Sect.  "Methods/experimental" using dropout. The others represent datasets were a certain parameter was omitted in the data generation processes such as lighting or textures. We also go a step further and texturize the cats and dogs in what is labelled as “texture subject” bar

We generate 7 new data sets, each omitting obstructors, textures, lighting, and sky boxes, keep subjects in default position as oppose to randomizing their pose, and omit breed variations, as well as making unrealistic subjects by applying random textures to them (e.g. wooden dogs). Results are sumarized in Fig. 4. The skies and the textures to the surrounding seem not to have significant effects on model accuracy. Texturizing the subjects intuitively should generalize better to different domain, but in the case of real world subjects, it seems to actually lower accuracy a bit. The largest significance was caused by the variety of different breeds. Initially we had 10 cat and 28 dog models. Limiting to just 1 of each reduced our accuracy by nearly 16%. What this shows is that the breed variation is the most important factor in learning differentiating features. This could also suggest that increasing the amount of breeds could increase model accuracy to levels near the real model. A surprising result however, was the fact that removing obstructors and keeping the subjects in their default upright poses increased model accuracy nearly 3%. This indicates that some poses could actually be hiding important features needed to classify each object. The obstructors on the other hand could be hiding parts of the subject or causing unnecessary shadows on the subject in ways where they might hide import classification information. This could also indicate that our real test data has a highly condensed distribution in terms of subjects being displayed very clearly in full, and generally being photographed staying still as oppose to being caught mid-action in a certain motion.

### Heatmaps

**Fig. 5:**
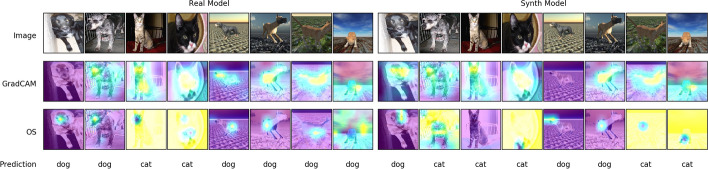
First 4 real and synthetic images in each category, and their subsequent analysis as to what each model is looking at to classify each image (real model on the left, synthetic on the right). Second row shows the GradCAM explainer while the third row utilizes Occulusion Sensitivity (OS), when testing for the cat class

Being mostly a black-box, neural network based methods are still susceptible to overfitting and other unforeseeable errors. For this fact, interpreting our model is another facet worth exploring before we can conclude anything concrete about its accuracy. For this, we apply two state-of-the-art heatmap based techniques to some images from both domains, and inspect which regions of the image our networks value most with respect to their final classification. Some non-handpicked examples can be seen in Fig. 5. First we apply GradCAM [22], to our images, and specify the cat category as our target class. For the most part, our network tends to focus on the facial features, including ears and eyes mostly to determine its prediction, in line with what we expect. Notice in Fig. 5 how each model tends to mistake dominant features in images outside its domain. We explore this further in Sect. "Domain transfer accuracy". The last row shows a heatmap of the output produced using the Occlusion Sensitivity method [23]. The results here seem to be more global, with a slight tendency to value a single eye of the subject.

### Domain transfer accuracy

**Fig. 6:**
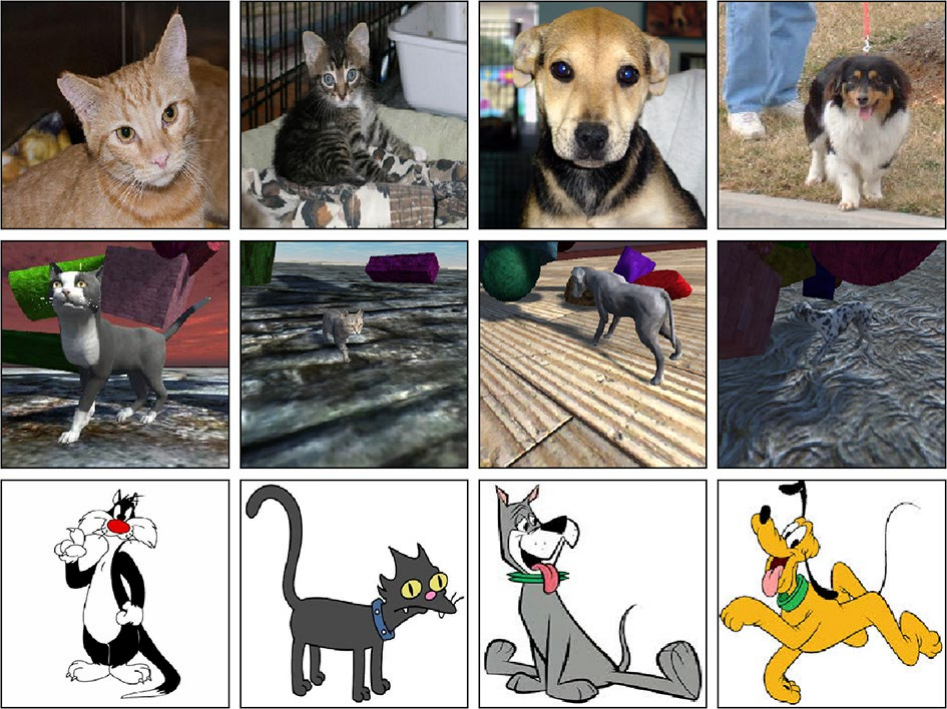
Example of cat and dog images shown to our models across different domains. First row is of real world domain images. The second row shows a computer generated 3D domain similar to video games. Third row is a new animated based domain unseen to both models during training. Cartoon images are intellectual property of Warner Bros., Fox Broadcasting Company, Hanna-Barbara Productions and Walt Disney Productions

**Fig. 7:**
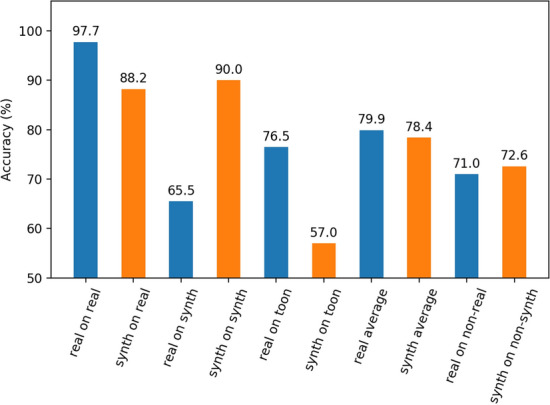
Optimal accuracy of the real and synthetic model across a variety of different domains

One property that a domain randomized data set may provide over a real one is an inherent ability to generalize to other domains. Since the real data set is obtained from a real world distribution and real world conditions, it is not surprising that it outperforms the synthetic set. However, it is of interest to see how such models will perform across domains it has not seen before. In this section, we test the best models we built thus far on a new small test set that consists of cartoon styled images of dogs and cats (see Fig.  6). In theory if our models are truly learning the correct set of features that separate cats from dogs, this task should produce reasonable results to the initial test set accuracy. Results from this test can be found in Fig.  7.

Contrary to our hypothesis, the synthetically trained model actually performs significantly worse on this new “Toon” domain vs the real model, by nearly 20%. However, what is interesting is despite the real model’s ability to transfer reasonable to the animated domain, it generalized very poorly when tested on the synthetic domain. This suggests that domain generalization is domain specific. A model may transfer well to another domain, while being useless on another. In the case of measuring out-of-domain accuracy, our synthetic model actually outperformed the real model on average, suggesting that domain randomization does in fact help with generalization.

### Increasing categories

So far, we have only looked at classification accuracy across 2 categories. It would also be of interest to see how our synthetically trained models perform as the number of categories increases. We combine the GRAZ-02 Dataset [24], with the MaviIntelligence Bike Dataset [25] and the StanfordAI Cars Dataset [26] for real world images of cars and bikes, and also incorporate 24 bike and 16 car 3D CAD models into our data generation process. We limit our studies to 4 categories as gathering 3D models for data generation purposes is a challenge in its own.

**Fig. 8:**
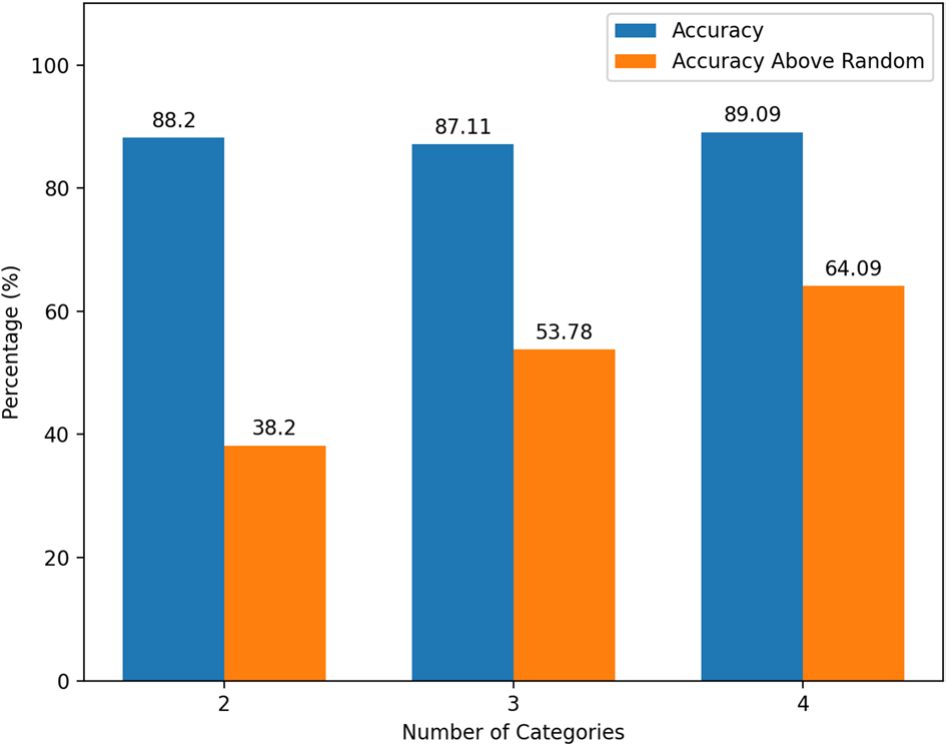
Accuracy of the model trained on synthetic data as the number of categories in the classification varies. The 2 category model trained between cat and dogs, the 3 category introduced bikes and the 4 category added the cars class

Our combined dataset now contains thousands of images of each class, where we again utilize a 80–20 train-test split. Results can be found in Fig. 8. Overall classification accuracy seemed to remain consistent moving up to 3 and 4 categories.. Alternatively, an accuracy above random guessing (AAR) is calculated which is computed by

$$\begin{aligned} AAR = Accuracy - \frac{1}{n}, \end{aligned}$$

where *n* is the number of categories. Looking at this measure which more accurately accounts for increases in labels, our networks’ performance rises with each subsequent category added. One might expect for the overall accuracy to begin to drop as the categories increase, while the AAR remains constant. However, this is not what we see, suggesting that models trained on synthetic data get better as the number of classes increases.

## Conclusion

Using domain randomization to generate a vast range of cat and dog images, we were able to train a classifier to identify photos or real world dogs and cats at an accuracy level of 85.26%. We were further able to increase the accuracy of the model (surprisingly) to 88.26% by actually limiting the posing randomization of the subjects. This was likely due to producing a distribution that was more similar to that of the real world test dataset. It was also found that the most important parameter to randomize is not surprisingly, the amount of breeds for each category.

Visual heatmaps suggest that our network is in fact looking at reasonable sections of the image to determine model predictions. Given that our model is trained on synthetic images, it generalized to out-of-training domain better than the model trained only on real world data, scoring an accuracy of 72.6% on a mixture of real and animation images.

Lastly, the synthetically generated data was able to maintain high accuracy when the number of categories for the classifier was increased.

Overall our results are in line with the current literature in other visual based tasks when it comes to the use of synthetic data in training neural networks. Our results show that despite not being able to outperform real world data based models, the computer generated data can achieve slightly worse results at the benefit of being easier and cheaper to attain and scale. Furthermore, as Schraml [20] mentioned, synthetic data should be used in unison with real data, as oppose to a replacement.

Since our initial goal was to compare the effectiveness directly of synthetic images vs real ones, we did not spend much time redesigning the network architecture itself, but focused specifically on finetuning the weights and dropout probabilities. It may very well be that the reality gap can be eliminated altogether, given some adjustments to the model itself. This would be an interesting area worth exploring, as to optimal network structure for the generalization of synthetic data to the real world domain.

## Declarations

### Availability of data and materials

The synthetic datasets generated during the current study have been made available at the Synthetic Image Dataset (Cats, Dogs, Bikes, Cars) Kaggle repository at https://www.kaggle.com/zarkonium/synthetic-image-dataset-cats-dogs-bikes-cars. The real image datasets used and/or analysed are available in the Dogs vs. Cats Kaggle, GRAZ-02, Maviintelligence and StanfordAI repositories [21, 24–26], at https://www.kaggle.com/c/dogs-vs-cats/data , https://www-old.emt.tugraz.at/~pinz/data/GRAZ_02/, http://maviintelligence.com/bicycle-image-dataset/, and https://ai.stanford.edu/~jkrause/cars/car_dataset.html respectively.
